# Prognostic Value of Inflammatory Indicators in Chronic Hepatitis B Patients With Significant Liver Fibrosis: A Multicenter Study in China

**DOI:** 10.3389/fphar.2021.653751

**Published:** 2021-11-10

**Authors:** Xiujuan Zhang, Yusheng Jie, Zemin Wan, Shanshan Lin, Yingxian Li, Ming Lin, Shuduo Wu, Xiaoju Wu, Meijie Shi, Huanming Xiao, Minling Cao, Jiao Gong, Xiaoling Chi

**Affiliations:** ^1^ Department of Hepatology Diseases, Guangdong Provincial Hospital of Chinese Medicine Second Affiliated Hospital of Guangzhou University of Chinese Medicine, Guangzhou, China; ^2^ Department of Infectious Diseases, Third Affiliated Hospital of Sun Yat-sen University, Guangzhou, China; ^3^ Department of Laboratory Medicine, Second Affiliated Hospital of Guangzhou University of Chinese Medicine, Guangzhou, China; ^4^ Department of Medical Education, Second Affiliated Hospital of Guangzhou University of Chinese Medicine, Guangzhou, China; ^5^ Department of Laboratory Medicine, Third Affiliated Hospital of Sun Yat-sen University, Guangzhou, China

**Keywords:** chronic hepatitis B, inflammatory indicators, liver fibrosis, diagnosis, predicting tool

## Abstract

Diagnosis of significant liver fibrosis is essential to facilitate the optimal treatment decisions and improve prognosis in patients with chronic hepatitis B (CHB). We aimed to evaluate the value of inflammatory indicators and construct a nomogram that effectively predicts significant liver fibrosis among CHB patients. 563 CHB patients from two centers in China from 2014 to 2019 were divided into three cohorts (development, internal validation, and independent validation cohorts), assigned into cases with significant fibrosis (liver fibrosis stages ≥2) and those without. Multiple biochemical and serological inflammatory indicators were investigated. Inflammatory indicators, Alanine aminotransferase (ALT) and aspartate aminotransferase (AST), were significantly associated with significant liver fibrosis in CHB patients but limited predictive performance, and then we combined them with prothrombin time activity percentage (PTA) and liver stiffness measurement (LSM) were identified by multivariate logistic regression analysis. Based on these factors, we constructed the nomogram with excellent performance. The area under the receiver operating characteristic curve (AUROC) for the nomogram in the development, internal validation, and independent validation cohorts were 0.860, 0.877, and 0.811, respectively. Our nomogram based on ALT and AST that had excellent performance in predicting significant fibrosis of CHB patients were constructed.

## Introduction

According to World Health Organization (WHO) statistics, hepatitis B virus (HBV) infection is a common global public health problem (296 million people were living with HBV infection and with 1.5 million new infections every year), particularly in Eastern Asia (WHO, 2020). HBV infection is moderately endemic in China. Persistent infection with HBV can progress to cirrhosis and hepatocellular carcinoma (HCC), which is one of the most frequent cancers in our country (El-Serag, 2012), have high morbidity and mortality (Marcellin, 2009). Most cases develop in the context of liver fibrosis (El-Serag, 2011). To reduce the disease adverse consequences of HBV infection, it may be very important to identify subjects with liver fibrosis or cirrhosis and treat them as soon as possible (Meng et al., 2015).

Liver fibrosis is characterized by an overall increase in the extracellular matrix, mainly produced by hepatic stellate cells (HSCs) (Xiao et al., 2020). It involves a phenotypic switch induced by numerous cell types in a cytokine-mediated inflammatory process. Early treatment for liver fibrosis can significantly reduce the inflammatory process and decrease the high mortality rate by successfully preventing cirrhosis and HCC. Therefore, early diagnosis of liver fibrosis is essential.

In clinical practice, imaging can easily identify cirrhosis, such as ultrasound, computed tomography (CT), and magnetic resonance imaging (MRI). However, accurately diagnosing the fibrosis stage is difficult without a liver biopsy, representing the gold standard for fibrosis staging in widely used guidelines for the prevention and treatment of CHB (Terrault et al., 2018; Gan et al., 2019). However, in clinical practice, the biopsy is the last strategy owing to its invasiveness, the risk of serious complications, and sampling limitations (sampling error and inter-assessor variation) (Rudolph et al., 2018). It is difficult and invasive for patients to undergo repeated biopsies for the dynamic staging of liver fibrosis.

According to the AASLD 2018 hepatitis B guidance, non-invasive methods could be used to assess the degree of fibrosis, such as LSM, APRI, and FIB-4, and they are used extensively. Still, reportedly, other non-invasive methods in predicting liver fibrosis, like FibroTest (consists of an algorithm of five fibrosis markers: alfa2-macroglobulin, apolipoproteinA1, haptoglobin, γ-glutamyl transferase(GGT), bilirubin), Hepascore (includes four biomarkers, hyaluronic acid, namely: alpha2-macroglobulin, GGT and bilirubin, as well as gender and age), FibroMeter (includes age, sex, platelets (PLT), alpha-2-macroglobulin, alanine aminotransferase (ALT), urea, prothrombin index (PI), GGT, and aspartate aminotransferase (AST)), Enhanced Liver Fibrosis test (consists of an algorithm of three fibrosis markers: hyaluronic acid, amino-terminal propeptide-of-type-III-collagen, tissue-inhibitor of matrix-metaloproteinase-1), etc.

The research (Friedrich-Rust M et al.) found the performance of diagnosis of significant liver fibrosis (F ≥ 2) for Enhanced Liver Fibrosis test and FibroTest, those AUROC for the diagnosis of significant fibrosis were 0.78 (95%CI:0.67–0.89) and 0.69 (95%-CI:0.57–0.82), respectively. In another study, Hepascore increased diagnostic accuracy in HBV with a mean obAUROC of 0.79 (95% CI, 0.75–0.83) (Huang et al., 2017a). Another research from Leroy V et al. found the AUROC of FibroMeter ranged from 0.75 to 0.84 superiority over FibroTest and Hepascore to diagnose significant fibrosis for significant fibrosis of CHB patients (Leroy et al., 2014).

Those scores have more excellent performance in predicting cirrhosis than predicting fibrosis (Friedrich-Rust et al., 2010; Xiao et al., 2015; Sonneveld et al., 2019). Moreover, some factors in those models were rarely used in routine clinical practice, leading to application limitations.

LSM is one of the most popular non-invasive measurements for evaluating liver stiffness and hepatic fibrosis (European Association for Study of Liver, 2015; Duan et al., 2020). Still, obesity, ascites, and limited operator experience inevitably result in unreliable and inaccurate measurement results (Castéra et al., 2010).

It is essential to build a tool to circumvent these drawbacks and accurately distinguish CHB patients with significant liver fibrosis (Chalasani et al., 2018; Vilar-Gomez and Chalasani, 2018). From the perspective of clinical practice, an ideal screening tool for significant fibrosis should be simple, non-invasive, convenient, and repeatable. In this study, we developed an accurately predictive nomogram and compared its performance with other commonly used predictors, including FIB-4 and APRI, to verify whether the nomogram was superior for clinical management.

## Patients and Methods

### Study Population and Inclusion and Exclusion Criteria

A retrospective cohort design was used to develop a nomogram to detect CHB-related significant fibrosis. All eligible patients at the Guangdong Province Traditional Chinese Medical Hospital between 2017 and 2019 and at the Third Affiliated Hospital of Sun Yat-sen University between 2014 and 2017 were enrolled. The inclusion criteria were as follows: CHB, which was defined as HBsAg positivity for >6 months; underwent liver biopsy, and had no missing value of multiple biochemical and serological inflammatory indicators. The exclusion criteria were as follows: alcoholic liver disease; liver cancer; co-infection with hepatitis C virus, hepatitis D virus, or human immunodeficiency virus; autoimmune liver diseases (such as autoimmune hepatitis, primary biliary cirrhosis, and primary sclerosing cholangitis); hereditary and metabolic liver diseases (such as Wilson’s disease); and missing data on important clinical parameters.

Ethical approval for the study was granted by the ethics committee of the Second Affiliated Hospital of Guangzhou University of Chinese Medicine, and the Ethics Commission of the Third Affiliated Hospital of SunYat-sen University.

### Liver Biopsy

Ultrasound-guided liver biopsy was performed using a quick-cut needle. Liver fibrosis staging (F0–F4) was carried out by a single experienced pathologist blinded to the clinical data according to the following Scheuer scoring system (Scheuer, 1991; Gan et al., 2019): F0 = no fibrosis, F1 = portal fibrosis without septa, F2 = perisinusoidal and portal/periportal fibrosis, F3 = bridging fibrosis, and F4 = highly suspicious or definite liver fibrosis was defined as fibrosis stages ≥2.

### Serum Markers

For each patient, a complete medical history, including blood test results (which had been conducted as routine clinical practice), were obtained via standard auto- mated laboratory methods and using commercially available kits following the manufacturer’s protocols. The data included α-fetoprotein (AFP, ng/ml), which was assessed using a Roche Cobas e602(Germany) analyzer. Additionally, liver function parameters, comprising alanine aminotransferase (ALT, U/L), aspartate aminotransferase (AST, U/L), γ-glutamyl transferase (GGT, U/L), albumin (ALB, g/L), ALB/globulin (GLB, A/G), total bilirubin (TBIL, μmol/L), direct bilirubin (DBIL, μmol/L), indirect bilirubin (IBIL, μmol/L), and total bile acid (TBA, μmol/L), were assessed using a Roche Cobas c702 analyzer (Germany). PLT count (10^9^/L) was evaluated using a Sysmex 5000 hematology analyzer (Japan). Prothrombin time (PT, s) was assessed using an STA-A-Evolution-II analyzer (France). Prothrombin time activity percentage (PTA, %) was determined based on the percentage of normal plasma yielding the same PT (in seconds), obtained from a curve constructed using serial dilutions (with saline) of pooled normal plasma (Robert and Chazouillères, 1996).

### Non-Invasive Predictors

Non-invasive fibrosis predictors (APRI (Wai et al., 2003) and FIB-4 (Sterling et al., 2006)) were calculated according to published formulas. APRI was calculated as follows: (AST [U/L]/upper limit of normal/PLT [10^9^/L]) × 100; FIB-4 was calculated as follows: (age [year]×AST [U/L])/(PLT [10^9^/L]×ALT [U/L]^1/2^).

### Liver Stiffness Measurement and Controlled Attenuation Parameter

LSM (kPa) and CAP (B/m) were assessed using a FibroScan device at each center by an experienced doctor blinded to the other clinical data. The data were considered valid if they met the following criteria (European Association for Study of Liver, 2015): >10 valid tests, success rate ≥60%, and interquartile range <30% of the median value of LSM, CAP.

### Statistical Analysis

The continuous variables are expressed as median (minimum, maximum), and the categorical variables are expressed as number and proportion. The continuous variables were analyzed using the Mann–Whitney U test, and the categorical variables were analyzed using the χ^2^ test in contingency tables.

To identify the relative importance of each feature, a least absolute shrinkage, and selection operator (LASSO) (Tibshirani, 2011) regression analysis were performed. The key predictors were then selected in the development cohort based on the variance inflation factor (VIF). After that, multivariate logistic regression analysis (forward stepwise method) was used to select the independent predictors included in the nomogram.

As described previously (Gong et al., 2020), a nomogram was built using the rms package in R software to provide a quantitative tool for clinical practice to predict individual probabilities of significant fibrosis. The performance of the nomogram was evaluated based on the area under the receiver operating characteristic curve (AUROC). The AUROC value was compared to the AUROC values for FIB-4 and APRI. A calibration curve was plotted, and the Hosmer–Lemeshow calibration test in R was conducted to compare the nomogram results with the liver fibrosis staging results using the Scheuer scoring system. Decision curve analysis (DCA) and clinical impact curve analysis (CICA) were used to evaluate the net benefit, namely, whether the nomogram does better (in terms of identifying significant fibrosis) than harm (in terms of biopsies). The nomogram based on the development cohort was used to calculate the total points and the associated probability of significant fibrosis for each patient in each validation cohort (internal and independent).

For all analyses, *p* < 0.05 was considered statistically significant. The statistical analyses were conducted using SPSS 26.0 and R software 4.0.

## Results

### Patient Characteristics

**FIGURE 1:**
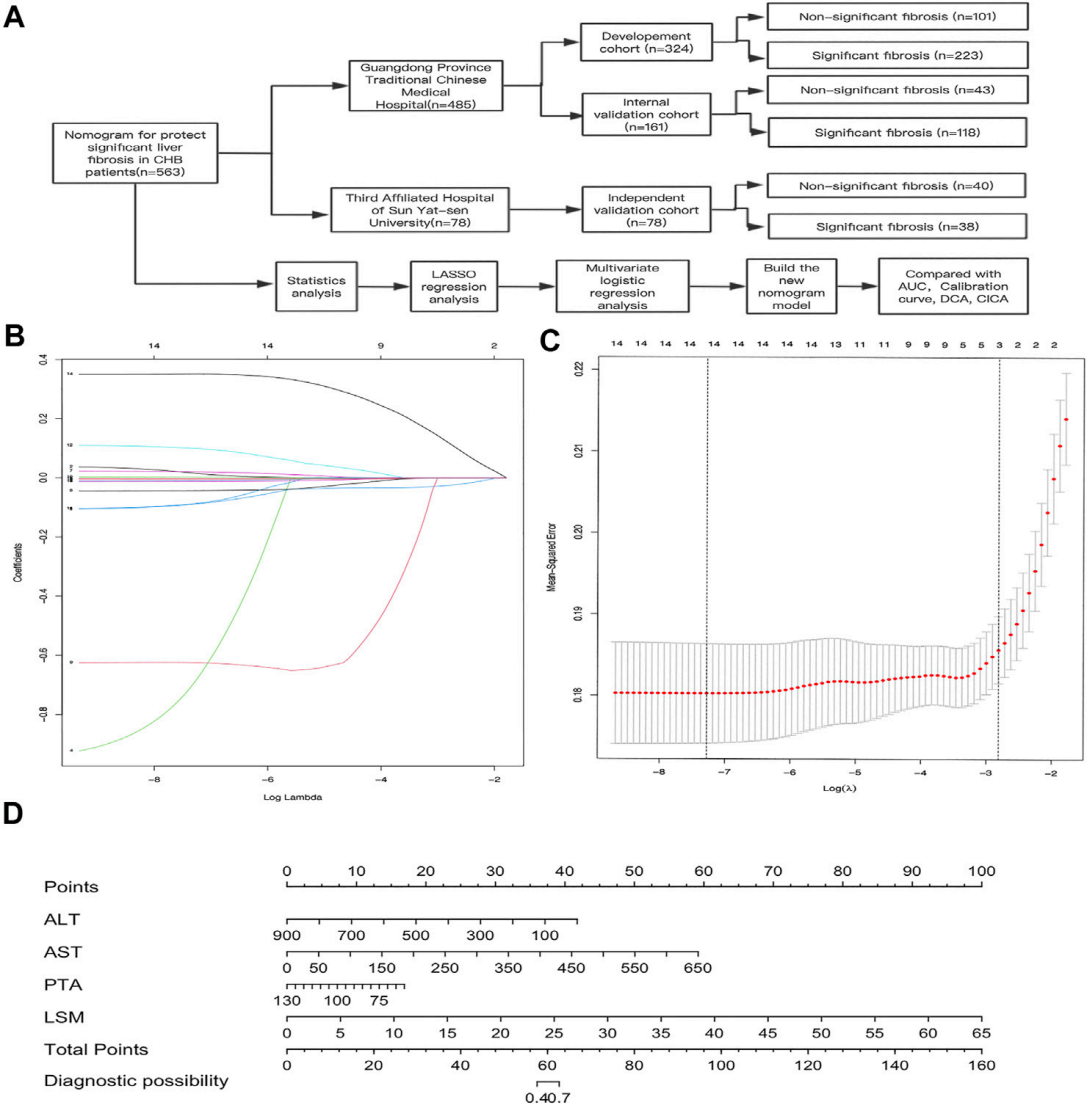
Construction of the AAPL nomogram (ALT, AST, PTA, and LSM) for predicting significant liver fibrosis in patients with CHB. **(A)** Flowchart of the study sample and AAPL nomogram construction. **(B)** Selection of fundamental clinical indicators using least absolute shrinkage and selection operator (LASSO) regression analysis. **(C)** Cross-validation plot for the penalty term. **(D)** AAPL nomogram for predicting significant fibrosis in patients with CHB.

A total of 563 CHB patients were enrolled in this study (Figure 1A). Of these, 485 (345 males and 140 females; aged from 16 to 70) were enrolled at the Guangdong Province Traditional Chinese Medical Hospital between 2017 and 2019. The number of cases with fibrosis stages F0, F1, F2, F3, and F4 were 3 (0.6%), 141 (29.1%), 218 (44.9%), 79 (16.3%), and 44 (9.1%), respectively (Table 1). Among these patients, 341 (70.3%) had significant fibrosis, and 144 (29.7%) did not. The patients were randomly divided into the development cohort (*n* = 324, F0 = 2(0.6%), F1 = 99(30.5%), F2 = 144(44.4%), F3 = 51(15.7%), F4 = 28(8.6%)) and the internal validation cohort (*n* = 161, F0 = 1(0.6%), F1 = 42(26%), F2 = 74(45.9%), F3 = 28(17.3%), F4 = 16(9.8%)) by random sampling (in SPSS 26.0). 223 (68.8%) patients had significant fibrosis in the development cohort, while 118 (73.3%) patients had significant fibrosis in the internal validation cohort. There were no significant differences in baseline characteristics between the two cohorts (all *p* > 0.05).

**TABLE 1:** Baseline characteristics of patients in the development and validation cohorts.

Variable	Development cohort	Internal validation cohort		Independent validation cohort	
	(*n* = 324)	(*n* = 161)	*p*-value	(*n* = 78)	*p*-value
Age (year)	38	38	0.489	40.5	0.4
	(16–70)	(20–67)		(10–61)	
Female (*n*, %)	98	42	0.395	18	0.265
	30.20%	30%		23.10%	
AFP (ng/ml)	3.77	4.12	0.922	NA	—
	(0.65–1,434)	(0.001–2,286)			
PLT (×10^9^/L)	200	199	0.803	NA	—
	(29–432)	(87–348)			
PT (s)	11.4	11.5	0.187	NA	—
	(9.5–14.5)	(10–15.8)			
PTA (%)	95.7	93.7	0.115	95.5	0.859
	(61–125.7)	(53–116.4)		(43–116)	
ALT (U/L)	36	36	0.325	36	0.903
	(5–831)	(8–985)		(5–831)	
AST (U/L)	29.5	30	0.342	27	0.412
	(11–645)	(10.6–584)		(17–1,482)	
ALB (g/L)	45.7	45.8	0.973	NA	—
	(22.7–56.2)	(23.6–54.6)			
A/G	1.6	1.6	0.716	NA	—
	(0.9–2.7)	(0.4–2.9)			
GGT (U/L)	29	32	0.239	NA	—
	(8–619)	(8–493)			
TBIL (μmol/L)	12.25	12.3	0.94	NA	—
	(2.8–44)	(4.4–47.8)			
DBIL (μmol/L)	4.9	4.8	0.987	NA	—
	(1.4–31.7)	(2.1–41.4)			
IBIL (μmol/L)	7.05	7.1	0.802	NA	—
	(0.9–30)	(2.3–35.8)			
TBA (μmol/L)	4.2	4.5	0.373	NA	—
	(0.5–128.5)	(0.7–240.4)			
LSM (kPa)	7.8	7.6	0.899	7.45	0.281
	(2.6–61.5)	(3.2–66.4)		(3.2–44.6)	
CAP (B/m)	221	221	0.715	NA	—
	(100–380)	(100–364)			
F0 (*n*, %)	2	1	—	14	—
	0.6%	0.6%		17.9	
F1 (*n*, %)	99	42	—	26	—
	30.5%	26%		33.3%	
F2 (*n*, %)	144	74	—	21	—
	44.4%	45.9%		26.9%	
F3 (*n*, %)	51	28	—	13	—
	15.7%	17.3%		16.6%	
F4 (*n*, %)	28	16	—	4	—
	8.6%	9.8%		5.1%	
Significant fibrosis (*n*, %)	223	118	0.343	38	0.001
	65.40%	34.60%		48.70%	

The continuous variables are expressed as median (minimum, maximum), and the categorical variables are expressed as number and proportion. The continuous variables were analyzed using the Mann–Whitney U test, and the categorical variables were analyzed using the χ^2^ test in contingency tables. AFP, α-fetoprotein; ALT, alanine aminotransferase; AST, aspartate aminotransferase; GGT, γ-glutamyl transferase; ALB, albumin; A/G, ALB/globulin (GLB); TBIL, total bilirubin; DBIL, direct bilirubin; IBIL, indirect bilirubin; TBA, total bile acid; PLT, platelets; PT, prothrombin time; PTA, prothrombin time activity percentage; LSM, liver stiffness measurement; CAP, controlled attenuation parameter. F0 = no fibrosis, F1 = portal fibrosis without septa, F2 = perisinusoidal and portal/periportal fibrosis, F3 = bridging fibrosis, and F4 = highly suspicious or definite cirrhosis. Significant liver fibrosis was defined as fibrosis stages ≥2. Differences were not statistically significant (*p* > 0.05) for any parameters among the three cohorts except for the percentage of patients with significant fibrosis between development and independent validation cohorts (*p* = 0.001).

A total of 78 patients (18 males and 60 females; aged from 10 to 61) were enrolled at the Third Affiliated Hospital of Sun Yat-sen University between 2014 and 2017. These patients comprised the independent validation cohort, including 38 (48.7%) patients with significant fibrosis and 40 (51.3%) patients without significant fibrosis. The number of cases in fibrosis stages F0, F1, F2, F3, and F4 were 14 (17.9%), 26 (33.3%), 21 (26.9%), 13 (16.7%), and 4 (5.1%), respectively. The baseline characteristics of the patients in the development and validation cohorts are summarized in Table 1.

### Nomogram Construction and Validation

In the development cohort, 15 fundamentals clinical indicators (AFP, PLT, PT, PTA, ALT, AST, ALB, A/G, GGT, TBIL, DBIL, IBIL, TBA, LSM, and CAP) were included in the LASSO regression analysis. We excluded irrelevant and redundant features. A total of 14 factors remained after the LASSO analysis (Figures 1B,C), and PT was excluded from further analysis owing to its high Variance Inflation Factor (VIF).

Based on these results, 13 variables (AFP, PLT, PTA, ALT, AST, ALB, A/G, GGT, DBIL, IBIL, TBA, LSM, and CAP) were used in the multivariate logistic regression analysis (forward stepwise method). The development cohort showed that four factors, comprising ALT, AST, PTA, and LSM (Table 2, *p* < 0.05), were identified as important independent risk factors for significant fibrosis. After that, we used these factors to build a nomogram, which was designated the AAPL nomogram, for predicting significant fibrosis (Figure 1D). The nomogram can be used as follows: obtain the scores for each predictive factor shown in the nomogram, calculate the sum of the scores to receive the total score and, lastly, use the total score to determine the predicted risk, which is the probability of significant fibrosis in patients with CHB.

**TABLE 2:** Logistic regression of significant fibrosis in the development cohort.

Variable	Odds ratio	95% CI		
		Lower	Upper	*p*-value
AFP	1.044	0.972	1.122	0.237
PLT	0.997	0.991	1.002	0.243
PTA	0.959	0.925	0.994	0.023
ALT	0.99	0.982	0.999	0.024
AST	1.021	1.003	1.04	0.026
ALB	0.95	0.864	1.046	0.297
A/G	0.513	0.154	1.7	0.275
GGT	1.001	0.993	1.01	0.786
DBIL	0.878	0.675	1.143	0.334
IBIL	1.129	0.984	1.296	0.084
TBA	0.987	0.964	1.011	0.299
LSM	1.428	1.253	1.627	<0.001
CAP	0.996	0.99	1.002	0.205

ALT, AST, PTA, and LSM were identified as independent risk factors for significant fibrosis (*p* < 0.05).

**FIGURE 2:**
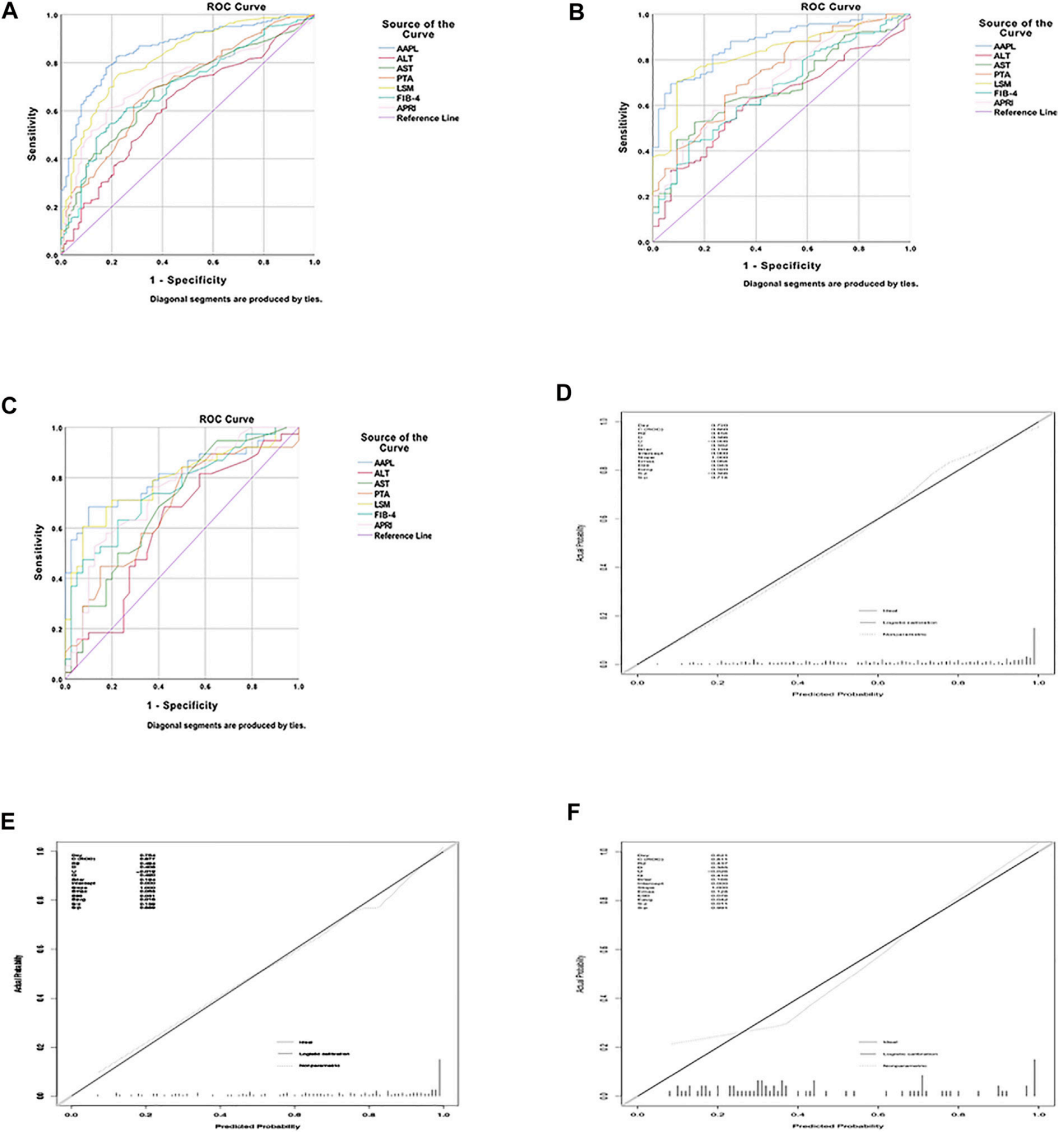
The area under the receiver operating characteristic (AUROC) curve and calibration curve for the AAPL nomogram (ALT, AST, PTA, and LSM). AUROC values of the AAPL nomogram for predicting significant fibrosis in patients with CHB in the **(A)** development cohort (AUROC = 0.86), **(B)** internal validation cohort (AUROC = 0.877), and **(C)** independent validation cohort (AUROC = 0.811). Calibration curve of the AAPL nomogram for predicting significant fibrosis in patients with CHB in the **(D)** development cohort, (E) internal validation cohort, and (F) independent validation cohort.

The AAPL nomogram had a significant and high AUROC (0.86 [95% confidence interval (CI), 0.818–0.902]) for distinguishing between individuals with significant fibrosis and those without significant fibrosis, with a high sensitivity of 0.825 and specificity of 0.782. Compared with the four independent risk factors alone and two other traditional predictors (FIB-4 and APRI), the AAPL nomogram had excellent performance in the three cohorts; the AUROC of the nomogram for predicting significant liver fibrosis in CHB patients was better than the AUROC of ALT (AUROC = 0.618, 95% CI 0.553–0.684), AST (AUROC = 0.687, 95% CI 0.627–0.747), PTA (AUROC = 0.701, 95% CI 0.239–0.359), LSM (AUROC = 0.821, 95% CI 0.773–0.87), FIB-4 (AUROC = 0.697, 95% CI 0.638–0.757), and APRI (AUROC = 0.725, 95% CI 0.669–0.781), as well as high sensitivity and specificity (Figure 2A and Table 3).

**TABLE 3:** The area under the receiver operating characteristic curve (AUROC) of the nomogram, independent risk factors (identified in the multivariate logistic regression analysis), and other scoring systems (FIB-4 and APRI) in the development cohort (cohort 1), internal validation cohort (cohort 2), and independent validation cohort (cohort 3).

	95% CI						
	Cohort	AUROC	Sensitivity	Specificity	*p*-value	Lower	Upper
AAPL	1	0.86	0.825	0.782	<0.001	0.818	0.902
ALT	1	0.618	0.646	0.584	<0.001	0.553	0.684
AST	1	0.687	0.695	0.634	<0.001	0.627	0.747
PTA	1	0.701	0.686	0.653	<0.001	0.239	0.359
LSM	1	0.821	0.753	0.772	0.03	0.773	0.87
FIB-4	1	0.697	0.543	0.812	<0.001	0.638	0.757
APRI	1	0.725	0.61	0.812	<0.001	0.669	0.781
AAPL	2	0.877	0.7	0.93	<0.001	0.823	0.931
ALT	2	0.624	0.627	0.628	0.016	0.533	0.715
AST	2	0.678	0.525	0.837	0.001	0.592	0.764
PTA	2	0.742	0.644	0.721	<0.001	0.175	0.341
LSM	2	0.83	0.703	0.907	<0.001	0.765	0.895
FIB-4	2	0.67	0.441	0.86	0.001	0.58	0.759
APRI	2	0.709	0.61	0.812	<0.001	0.624	0.795
AAPL	3	0.811	0.684	0.9	<0.001	0.712	0.909
ALT	3	0.609	0.684	0.575	0.099	0.482	0.735
AST	3	0.693	0.947	0.35	0.003	0.577	0.81
PTA	3	0.678	0.842	0.5	0.007	0.202	0.443
LSM	3	0.789	0.605	0.925	<0.001	0.685	0.892
FIB-4	3	0.75	0.632	0.775	<0.001	0.641	0.858
APRI	3	0.745	0.579	0.825	<0.001	0.635	0.854

AAPL is a new nomogram for predict significant liver fibrosis that consisted of ALT, AST, PTA, and LSM.

Furthermore, to confirm the broad applicability of the nomogram, we assessed its performance in two validation cohorts. The AUROC of the internal and independent validation cohorts were 0.877 (95% CI, 0.823–0.931) and 0.811 (95% CI, 0.712–0.909), respectively, higher than that of the four separate risk factors, FIB-4, and APRI (Figures 2B,C and Table 3). The calibration curve of the AAPL nomogram for predicting significant fibrosis demonstrated the excellent agreement between the nomogram predictions and actual observations in the development cohort (*p* = 0.714), internal validation cohort (*p* = 0.889), and independent validation cohort (*p* = 0.991) (Figures 2D–F).

**FIGURE 3:**
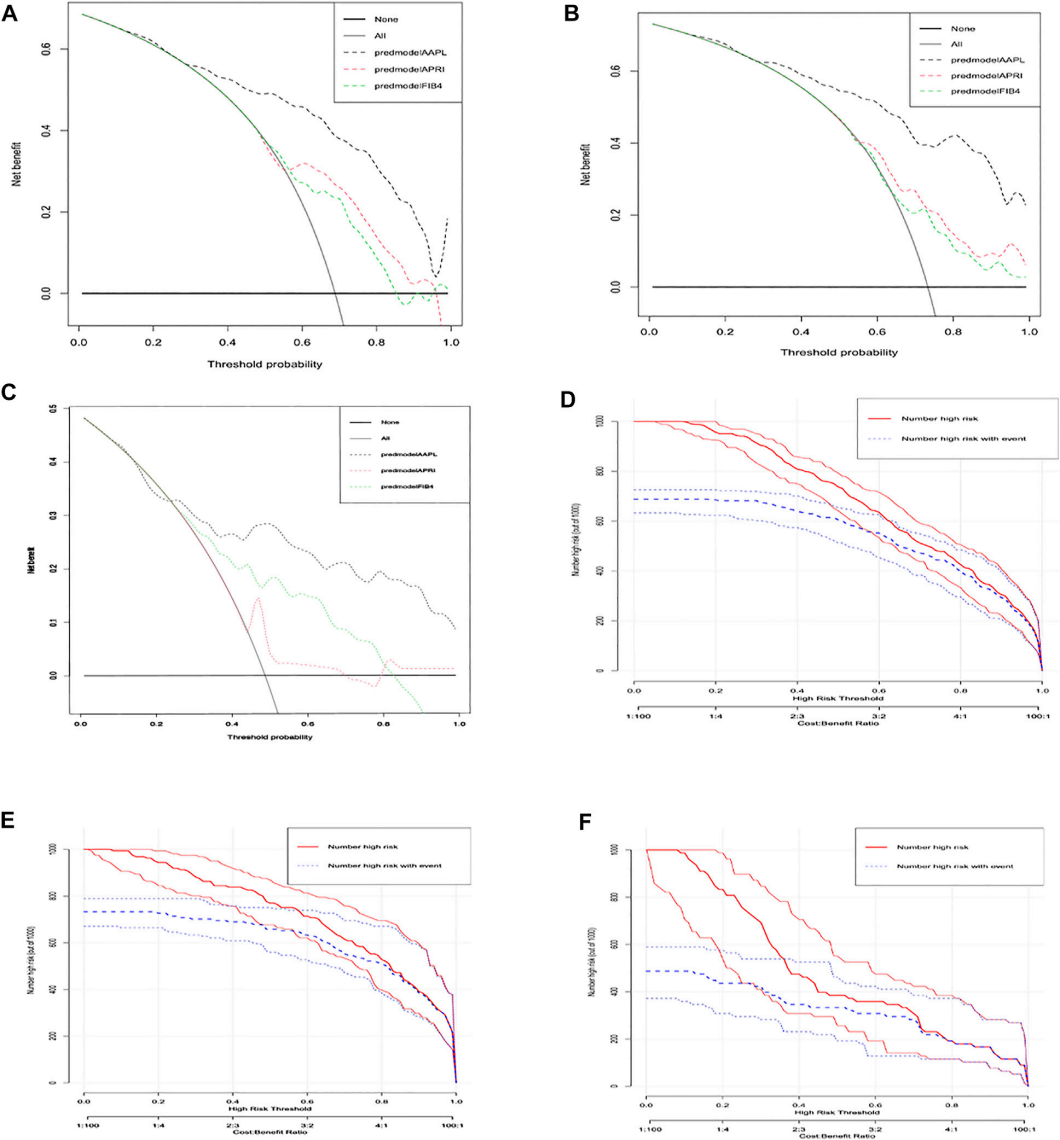
Decision curve analysis (DCA) and clinical impact curve analysis (CICA) of the AAPL nomogram (ALT, AST, PTA, and LSM). DCA of the AAPL nomogram for predicting significant fibrosis in patients with CHB in the **(A)** development cohort, **(B)** internal validation cohort, and **(C)** independent validation cohort. CICA of the AAPL nomogram for predicting significant fibrosis in patients with CHB in the **(D)** development cohort, **(E)** internal validation cohort, and **(F)** independent validation cohort.

To evaluate the clinical applicability of the new predictive nomogram, DCA and CICA were performed. The DCA (Figures 3A–C) and CICA (Figures 3D–F) showed that the nomogram had a superior overall net benefit within the vast and practical ranges of impacted patient outcomes and threshold probabilities.

## Discussion

Accurate assessment of the liver fibrosis stage is essential to determine whether CHB patients need to receive anti-viral treatment, as recommended by the American Association for the Study of Liver Diseases (AASLD) (Terrault et al., 2018). Liver biopsy is costly and invasive. Therefore, a tool to screen for and diagnose CHB patients with significant liver fibrosis in an acceptable, accurate, and dynamic manner is urgently needed (Ellis and Mann, 2012).

LSM is one of the best non-invasive measures of liver fibrosis, and it is recommended by several guidelines (Li et al., 2016; Terrault et al., 2018). However, several studies showed that its excellent performance abruptly decreased in the presence of ascites, obesity, and limited operator experience (Castéra et al., 2010; Udompap et al., 2020). We found good AUROC for LSM in all three cohorts (development cohort = 0.821, internal cohort = 0.83, and independent cohort = 0.789). However, to improve the prediction of significant fibrosis, we combined LSM with several other factors.

This study found that ALT, AST, PTA, and LSM on admission were associated with a higher risk of significant liver fibrosis by LASSO and multivariate logistic regression analysis. After that, we developed an accurate nomogram for predicting significant fibrosis. According to our study, ALT is an independent risk factor for significant fibrosis (odds ratio [OR] = 0.99, *p* = 0.024). The increased ALT in CHB patients occurs due to progressive liver inflammation (mainly induced by HSCs and immune cells) (Lee and Friedman, 2011; Xiao et al., 2020), which causes and accelerates fibrosis. However, the range of AUROC values for ALT was 0.619–0.624 in the three cohorts.

Similarly, Li et al. concluded that although ALT is commonly used to assess the staging of liver inflammation, it has a limited association with fibrosis (Li et al., 2018). On the other hand, ALT and AST are common indicators of liver inflammation and, in certain liver diseases, the former is used to assess the stage of liver fibrosis progression (Gonzalez et al., 2015). In addition to ALT, we identified AST as an independent risk factor for significant fibrosis (OR = 1.04, *p* = 0.026), and the range of AUROC was 0.678–0.693 across the three cohorts, which was higher than for ALT.

In CHB patients, PT is a measurement of synthetic liver function, failure, and cirrhosis. PTA is defined as the percentage of normal plasma that yields the same PT in seconds, which is more stable than PT in patients with liver disease (Robert and Chazouillères, 1996; Takikawa et al., 2014). After LASSO analysis and multivariate regression analysis, we identified PTA as one of the independent risk factors for significant liver fibrosis (OR = 0.959, *p* = 0.023), and the AUROC were 0.678–0.742 across the three cohorts.

Furthermore, our AAPL nomogram (composed of ALT, AST, PTA, and LSM) for predicting significant fibrosis among CHB patients had significantly higher specificity and sensitivity than the individual factors alone. The calibration plot demonstrated the excellent agreement between the AAPL nomogram predictions of significant fibrosis and the actual observations in the three cohorts. More importantly, CICA and DCA indicated that the nomogram had a superior overall net benefit within the wide and practical ranges of impacted patient outcomes and threshold probabilities.

Several traditional non-invasive predictors such as FIB-4 and APRI are commonly used to predict liver cirrhosis. They have exhibited outstanding performance in chronic hepatitis C virus patients (Li et al., 2019; Said et al., 2019). Although these predictors have decreased the need for liver biopsy in some patients, their performance has not been reproduced in CHB patients, especially in significant liver fibrosis patients (Xiao et al., 2015). In this study, the AUROC of the nomogram was higher than that of FIB-4 and APRI for predicting significant liver fibrosis, implying that the nomogram had a more excellent value than FIB-4 and APRI for predicting significant fibrosis in CHB patients. Consistent with our results, Huang et al. found that the AUROC of FIB-4 and APRI for predicting significant fibrosis were low (Huang et al., 2017b).

Our research has several advantages. First, we included CHB patients from multiple centers, obtaining a relatively large sample size, a total of 563 CHB patients. Second, the results from the development cohort were validated using internal and independent cohorts to confirm the robustness of our conclusion. Third, although the predictive nomogram consisted of only four factors, the values of these factors can be easily and non-invasively obtained in routine clinical practice. Lastly, the performance of the nomogram was excellent and efficient for clinical application, with a better AUROC than that of other established scoring systems (FIB-4 and APRI). This nomogram is a quantitative predictive tool that could facilitate decisions related to patient treatment.

There were several imitations. First, we enrolled 563 CHB in this retrospective study, and the performance of the nomogram in patients with other liver diseases or other populations outside of China might be affected by differences in patient factors and healthcare provision. To obtain a homogeneous study group, we only enrolled CHB patients, which might have led to selection bias. However, strict selection criteria can increase the accuracy of the diagnostic prediction and decrease the influence of other factors. Second, this is a retrospective study without any longitudinal follow-up data, so there was no assessment of the performance of the nomogram for dynamic prediction of liver fibrosis stage over time in CHB patients. The following research plan is to recruit patients for prospective studies to verify the effect of the model.

Moreover, some inflammation factors were not included in our research, such as CRP. That was often considered acute phase reactants (Gedik et al., 2007). However, in the present study, we mainly focused on the relationship between fibrosis and chronic inflammation. Hakki Yilmaz et al. found no correlation between fibrosis states and CRP in chronic hepatitis B (Yilmaz et al., 2015). The study is a retrospective study with large missing values of CRP (more than 95%). Hence, it was not included in the analysis. We will conduct further prospective studies, collecting CRP and other factors in the future, further uncover the association between CRP and significant liver fibrosis in CHB patients.

In summary, according to our study, inflammatory indicators are significantly associated with liver fibrosis in CHB patients. The predictive performance of the inflammatory markers ALT and AST was limited. Still, we combined them with LSM and PTA to establish a predictive nomogram for predicting individuals with significant liver fibrosis. Using this nomogram, we can accurately predict the risk of significant fibrosis in CHB patients in China, which will help to improve screening, early identification, and treatment selection for these high-risk patients.

## Data Availability

All data generated or analyzed during this study are included in this published article.

## References

[B1] Castéra L. Foucher J. Bernard P. H. Carvalho F. Allaix D. Merrouche W. (2010). Pitfalls of Liver Stiffness Measurement: a 5-year Prospective Study of 13,369 Examinations. Hepatology 51, 828–835. 10.1002/hep.23425 20063276

[B2] Chalasani N. Younossi Z. Lavine J. E. Charlton M. Cusi K. Rinella M. (2018). The Diagnosis and Management of Nonalcoholic Fatty Liver Disease: Practice Guidance from the American Association for the Study of Liver Diseases. Hepatology 67, 328–357. 10.1002/hep.29367 28714183

[B3] Duan W. J. Wang X. Z. Ma A. L. Shang J. Nan Y. M. Gao Z. L. (2020). Multicenter Prospective Study to Validate a New Transient Elastography Device for Staging Liver Fibrosis in Patients with Chronic Hepatitis B. J. Dig. Dis. 21, 519–525. 10.1111/1751-2980.12924 32700794

[B4] El-Serag H. B. (2012). Epidemiology of Viral Hepatitis and Hepatocellular Carcinoma. Gastroenterology 142, 1264–e1. 10.1053/j.gastro.2011.12.061 22537432 PMC3338949

[B5] El-Serag H. B. (2011). Hepatocellular Carcinoma. N. Engl. J. Med. 365, 1118–1127. 10.1056/NEJMra1001683 21992124

[B6] Ellis E. L. Mann D. A. (2012). Clinical Evidence for the Regression of Liver Fibrosis. J. Hepatol. 56, 1171–1180. 10.1016/j.jhep.2011.09.024 22245903

[B7] European Association for Study of Liver (2015). EASL-ALEH Clinical Practice Guidelines: Non-invasive Tests for Evaluation of Liver Disease Severity and Prognosis. J. Hepatol. 63, 237–264. 10.1016/j.jhep.2015.04.006 25911335

[B8] Friedrich-Rust M. Rosenberg W. Parkes J. Herrmann E. Zeuzem S. Sarrazin C. (2010). Comparison of ELF, FibroTest and FibroScan for the Non-invasive Assessment of Liver Fibrosis. BMC Gastroenterol. 10, 103. 10.1186/1471-230X-10-103 20828377 PMC2944336

[B9] Gan Z. Bing Z. Zhi Z. (2019). The Guidelines of Prevention and Treatment for Chronic Hepatitis B (2019 Version). Chin. Soc. Infect. Dis. CMA, Chin. Soc. Hepatol. CMA 27, 938–961. 10.3760/cma.j.issn.1007-3418.2019.12.007 PMC12813922 31941257

[B10] Gedik M. Ozekinci T. Ozbek E. Atmaca S. Yilmaz S. (2007). Lack of Correlation between CRP and Hepatitis B Viral Load in Serum of Patients with Chronic HBV. J. Infect. 54, 204. 10.1016/j.jinf.2006.04.009 16780953

[B11] Gong J. Ou J. Qiu X. Jie Y. Chen Y. Yuan L. (2020). A Tool for Early Prediction of Severe Coronavirus Disease 2019 (COVID-19): A Multicenter Study Using the Risk Nomogram in Wuhan and Guangdong, China. Clin. Infect. Dis. 71, 833–840. 10.1093/cid/ciaa443 32296824 PMC7184338

[B12] Gonzalez F. A. Van den Eynde E. Perez-Hoyos S. Navarro J. Curran A. Burgos J. (2015). Liver Stiffness and Aspartate Aminotransferase Levels Predict the Risk for Liver Fibrosis Progression in Hepatitis C virus/HIV-Coinfected Patients. HIV Med. 16, 211–218. 10.1111/hiv.12197 25234826

[B13] Huang R. Wang G. Tian C. Liu Y. Jia B. Wang J. (2017). Gamma-glutamyl-transpeptidase to Platelet Ratio Is Not superior to APRI,FIB-4 and RPR for Diagnosing Liver Fibrosis in CHB Patients in China. Sci. Rep. 7, 8543. 10.1038/s41598-017-09234-w 28819319 PMC5561053

[B14] Huang Y. Adams L. A. Joseph J. Bulsara M. K. Jeffrey G. P. (2017). The Ability of Hepascore to Predict Liver Fibrosis in Chronic Liver Disease: a Meta-Analysis. Liver Int. 37, 121–131. 10.1111/liv.13116 26991726

[B15] Lee U. E. Friedman S. L. (2011). Mechanisms of Hepatic Fibrogenesis. Best Pract. Res. Clin. Gastroenterol. 25, 195–206. dio:. 10.1016/j.bpg.2011.02.005 21497738 PMC3079877

[B16] Leroy V. Sturm N. Faure P. Trocme C. Marlu A. Hilleret M. N. (2014). Prospective Evaluation of FibroTest®, FibroMeter®, and HepaScore® for Staging Liver Fibrosis in Chronic Hepatitis B: Comparison with Hepatitis C. J. Hepatol. 61, 28–34. 10.1016/j.jhep.2014.02.029 24631902

[B17] Li Q. Zhou Y. Huang C. Li W. Chen L. (2018). A Novel Diagnostic Algorithm to Predict Significant Liver Inflammation in Chronic Hepatitis B Virus Infection Patients with Detectable HBV DNA and Persistently normal Alanine Transaminase. Sci. Rep. 8, 15449. 10.1038/s41598-018-33412-z 30337643 PMC6193950

[B18] Li X. Xu H. Gao P. (2019). Fibrosis Index Based on 4 Factors (FIB-4) Predicts Liver Cirrhosis and Hepatocellular Carcinoma in Chronic Hepatitis C Virus (HCV) Patients. Med. Sci. Monit. 25, 7243–7250. 10.12659/MSM.918784 31558693 PMC6784625

[B19] Li Y. Huang Y. S. Wang Z. Z. Yang Z. R. Sun F. Zhan S. Y. (2016). Systematic Review with Meta-Analysis: the Diagnostic Accuracy of Transient Elastography for the Staging of Liver Fibrosis in Patients with Chronic Hepatitis B. Aliment. Pharmacol. Ther. 43, 458–469. 10.1111/apt.13488 26669632

[B20] Marcellin P. (2009). Hepatitis B and Hepatitis C in 2009. Liver Int. 29 Suppl 1 (S1), 1–8. 10.1111/j.1478-3231.2008.01947.x 19207959

[B21] Meng F. Zheng Y. Zhang Q. Mu X. Xu X. Zhang H. (2015). Noninvasive Evaluation of Liver Fibrosis Using Real-Time Tissue Elastography and Transient Elastography (FibroScan). J. Ultrasound Med. 34, 403–410. 10.7863/ultra.34.3.403 25715361

[B22] Robert A. Chazouillères O. (1996). Prothrombin Time in Liver Failure: Time, Ratio, Activity Percentage, or International Normalized Ratio. Hepatology 24, 1392–1394. 10.1053/jhep.1996.v24.pm0008938167 8938167

[B23] Rudolph B. Bjorklund N. Ovchinsky N. Kogan-Liberman D. Perez A. Liszewski M. (2018). Methods to Improve the Noninvasive Diagnosis and Assessment of Disease Severity in Children with Suspected Nonalcoholic Fatty Liver Disease (NAFLD): Study Design. Contemp. Clin. Trials 75, 51–58. 10.1016/j.cct.2018.10.012 30401631 PMC6249118

[B24] Said M. Soliman Z. Daebes H. M El-Nahaas S. (2019). Real Life Application of FIB-4 & APRI during Mass Treatment of HCV Genotype 4 with Directly Acting Anti-viral Agents in Egyptian Patients, an Observational Study. Expert Rev. Gastroenterol. Hepatol. 13, 1189–1195. 10.1080/17474124.2019.1690990 31702417

[B25] Scheuer P. J. (1991). Classification of Chronic Viral Hepatitis: a Need for Reassessment. J. Hepatol. 13, 372–374. 10.1016/0168-8278(91)90084-o 1808228

[B26] Sonneveld M. J. Brouwer W. P. Chan H. L. Piratvisuth T. Jia J. D. Zeuzem S. (2019). Optimisation of the Use of APRI and FIB-4 to Rule Out Cirrhosis in Patients with Chronic Hepatitis B: Results from the SONIC-B Study. Lancet Gastroenterol. Hepatol. 4, 538–544. 10.1016/S2468-1253(19)30087-1 30975477

[B27] Sterling R. K. Lissen E. Clumeck N. Sola R. Correa M. C. Montaner J. (2006). Development of a Simple Noninvasive index to Predict Significant Fibrosis in Patients with HIV/HCV Coinfection. Hepatology 43, 1317–1325. 10.1002/hep.21178 16729309

[B28] Takikawa Y. Harada M. Wang T. Suzuki K. (2014). Usefulness and Accuracy of the International Normalized Ratio and Activity Percent of Prothrombin Time in Patients with Liver Disease. Hepatol. Res. 44, 92–101. 10.1111/hepr.12093 23521497

[B29] Terrault N. A. Lok A. S. F. McMahon B. J. Chang K. M. Hwang J. P. Jonas M. M. (2018). Update on Prevention, Diagnosis, and Treatment of Chronic Hepatitis B: AASLD 2018 Hepatitis B Guidance. Hepatology 67, 1560–1599. 10.1002/hep.29800 29405329 PMC5975958

[B30] Tibshirani R. (2011). Regression Shrinkage and Selection via the Lasso: a Retrospective. J. R. Stat. Soc. Ser. B-Statistical Methodol. 73, 273–282. dio. 10.1111/j.1467-9868.2011.00771.x

[B31] Udompap P. Sukonrut K. Suvannarerg V. Pongpaibul A. Charatcharoenwitthaya P. (2020). Prospective Comparison of Transient Elastography, point Shear Wave Elastography, APRI and FIB-4 for Staging Liver Fibrosis in Chronic Viral Hepatitis. J. Viral Hepat. 27, 437–448. 10.1111/jvh.13246 31799740

[B32] Vilar-Gomez E. Chalasani N. (2018). Non-invasive Assessment of Non-alcoholic Fatty Liver Disease: Clinical Prediction Rules and Blood-Based Biomarkers. J. Hepatol. 68, 305–315. 10.1016/j.jhep.2017.11.013 29154965

[B33] Wai C. T. Greenson J. K. Fontana R. J. Kalbfleisch J. D. Marrero J. A. Conjeevaram H. S. (2003). A Simple Noninvasive index Can Predict Both Significant Fibrosis and Cirrhosis in Patients with Chronic Hepatitis C. Hepatology 38, 518–526. 10.1053/jhep.2003.50346 12883497

[B34] WHO (2020). Hepatitis B.

[B35] Xiao G. Yang J. Yan L. (2015). Comparison of Diagnostic Accuracy of Aspartate Aminotransferase to Platelet Ratio index and Fibrosis-4 index for Detecting Liver Fibrosis in Adult Patients with Chronic Hepatitis B Virus Infection: a Systemic Review and Meta-Analysis. Hepatology 61, 292–302. 10.1002/hep.27382 25132233

[B36] Xiao W. Lu M. H. Rong P. F. Zhang H. Y. Gong J. Peng Y. Q. (2020). 11β-hydroxysteroid D-ehydrogenase-1 I-s A-ssociated with the A-ctivation of H-epatic S-tellate C-ells in the D-evelopment of H-epatic F-ibrosis. Mol. Med. Rep. 22, 3191–3200. 10.3892/mmr.2020.11423 32945429 PMC7453648

[B37] Yilmaz H. Yalcin K. S. Namuslu M. Celik H. T. Sozen M. Inan O. (2015). Neutrophil-Lymphocyte Ratio (NLR) Could Be Better Predictor Than C-Reactive Protein (CRP) for Liver Fibrosis in Non-alcoholic Steatohepatitis(NASH). Ann. Clin. Lab. Sci. 45, 278–286. 26116591

